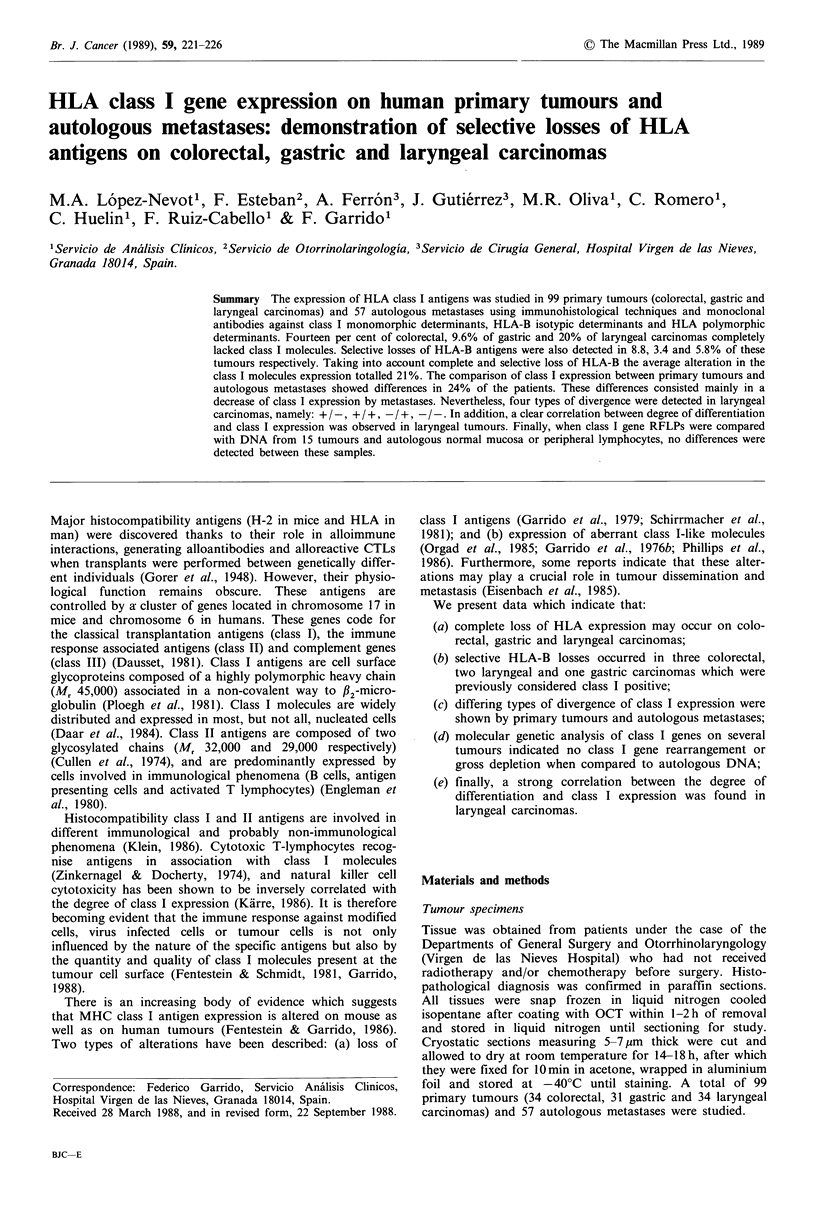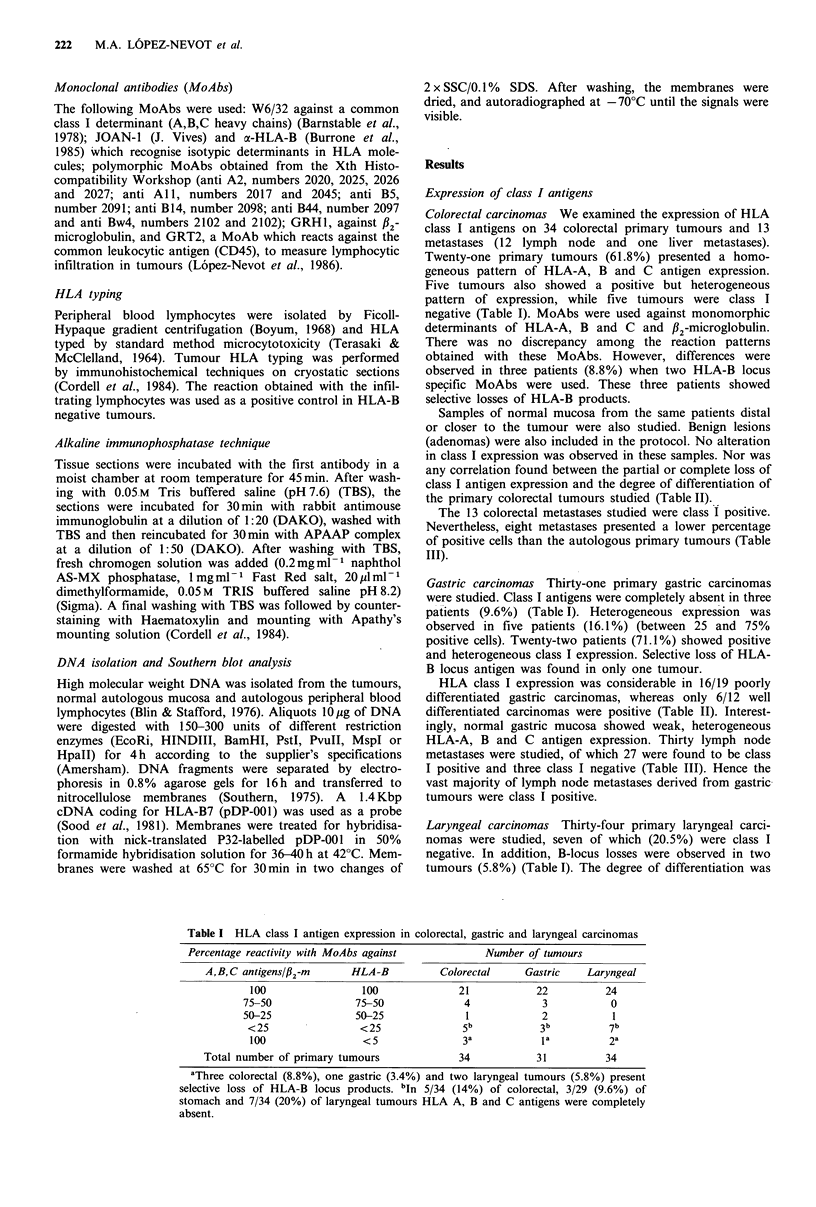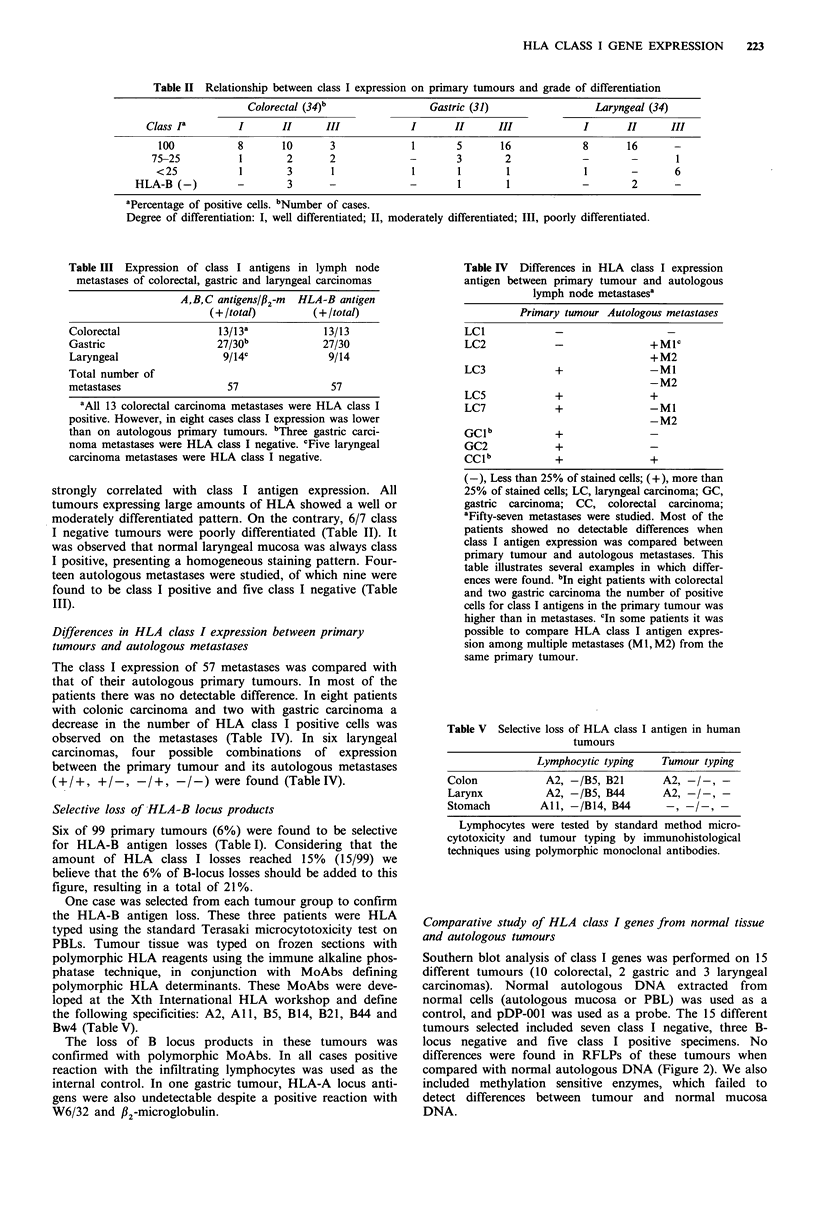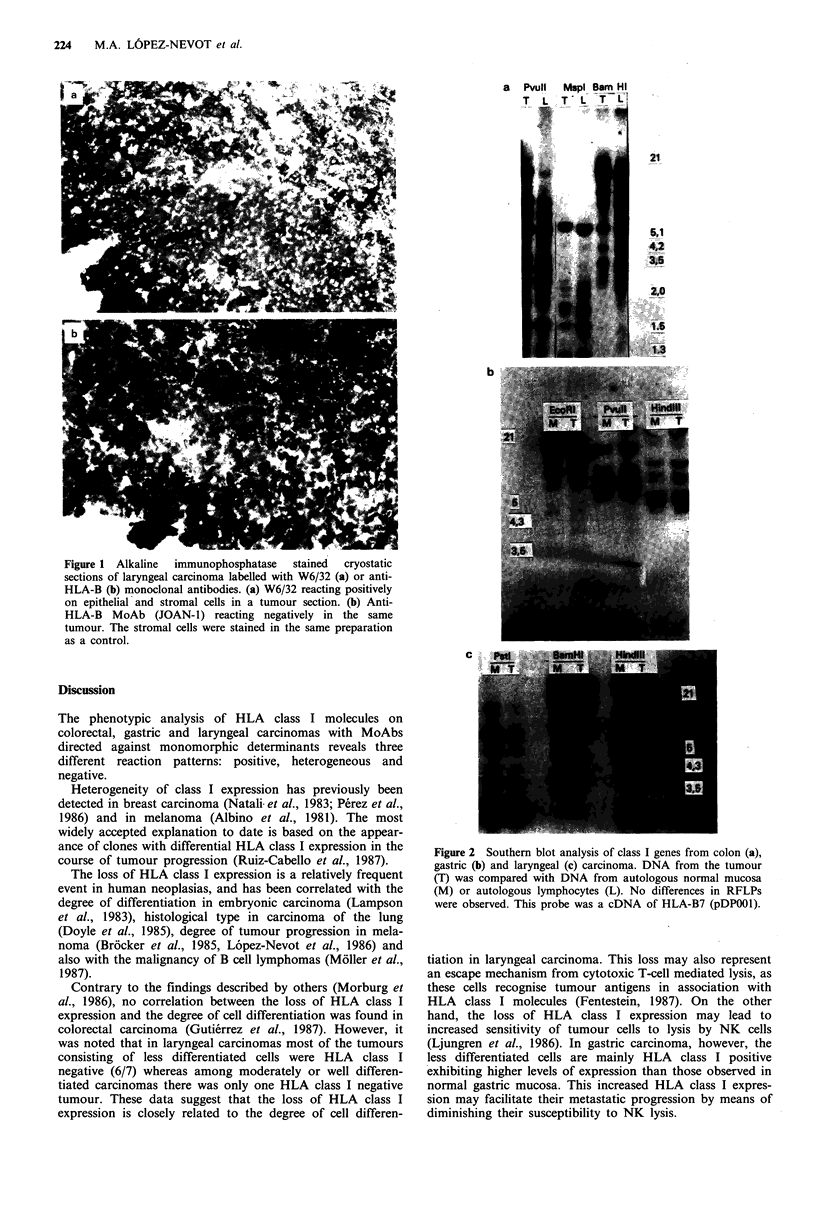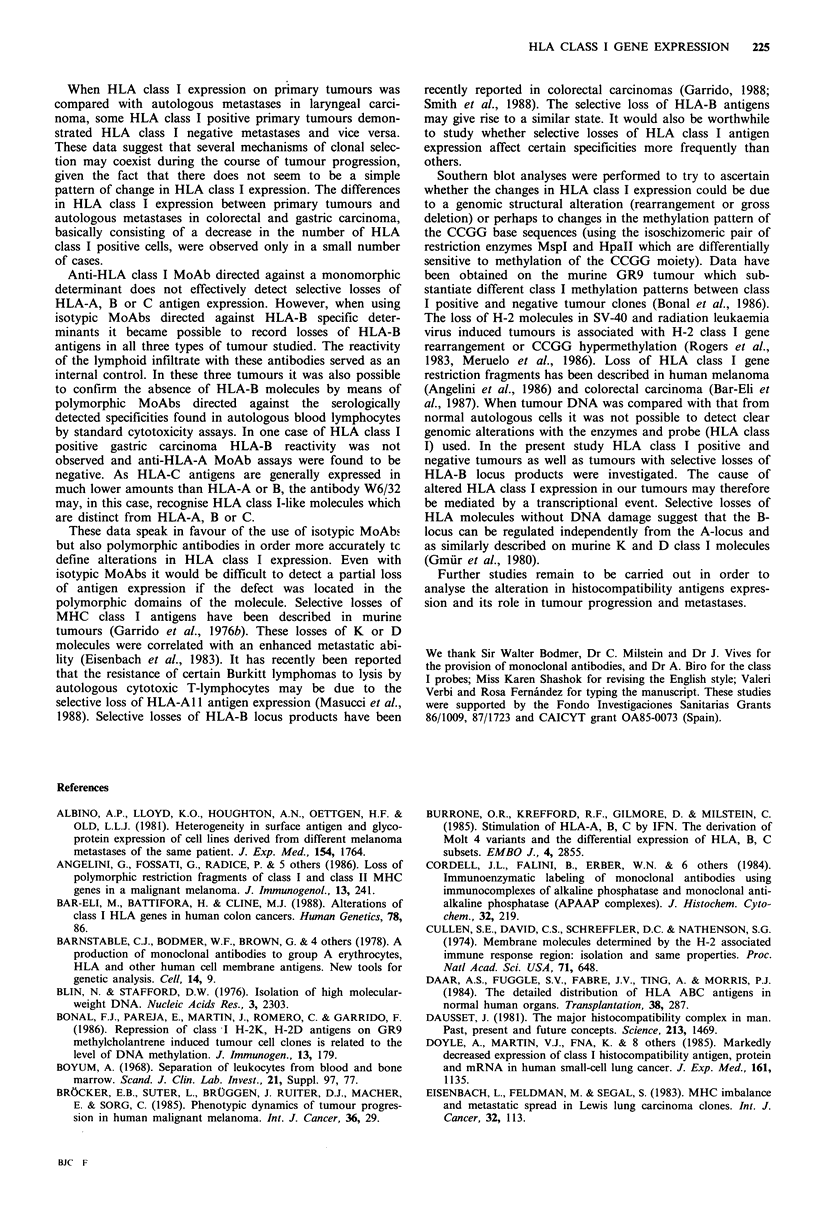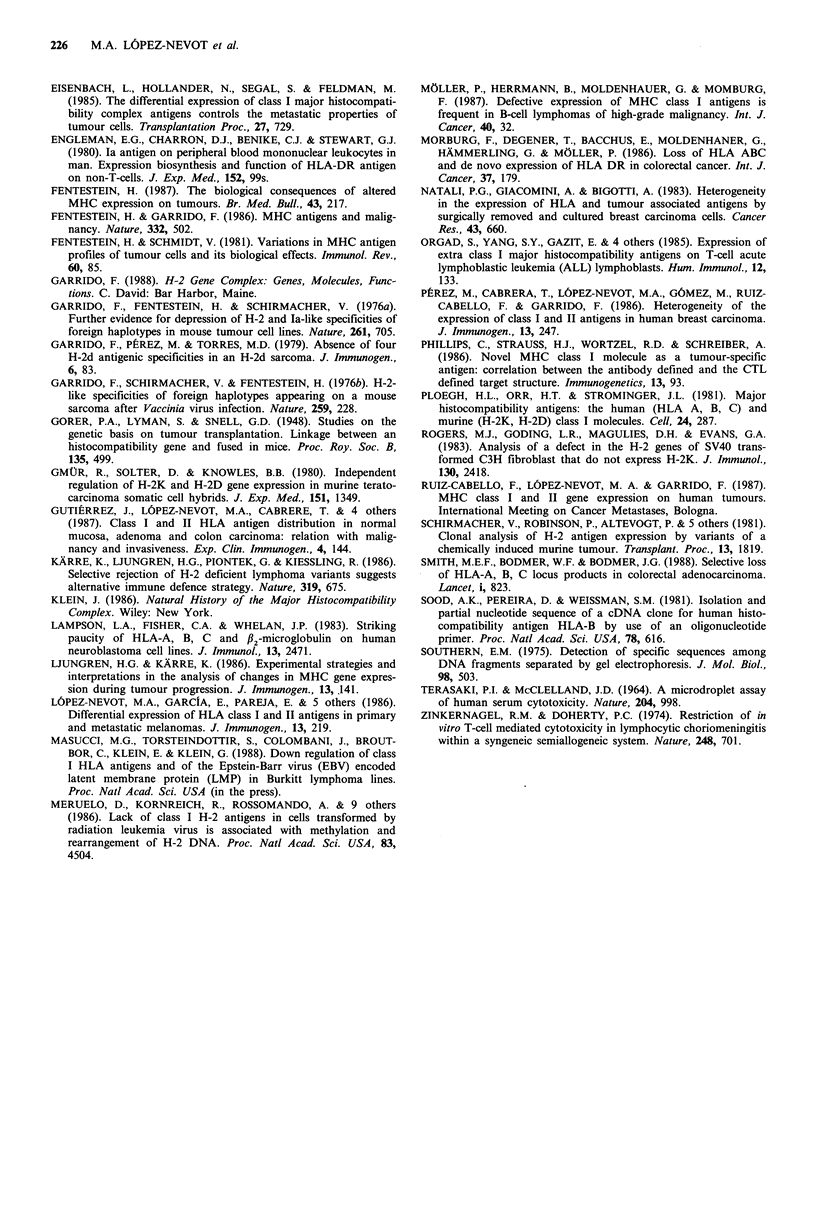# HLA class I gene expression on human primary tumours and autologous metastases: demonstration of selective losses of HLA antigens on colorectal, gastric and laryngeal carcinomas.

**DOI:** 10.1038/bjc.1989.45

**Published:** 1989-02

**Authors:** M. A. LÃ³pez-Nevot, F. Esteban, A. FerrÃ³n, J. GutiÃ©rrez, M. R. Oliva, C. Romero, C. Huelin, F. Ruiz-Cabello, F. Garrido

**Affiliations:** Servicio de AnÃ¡lisis ClÃ­nicos, Hospital Virgen de las Nieves, Granada, Spain.

## Abstract

**Images:**


Br. J. Cancer (1989), 59, 221–226 © The Macmillan Press Ltd., 1989

### HLA class I gene expression on human primary tumours and autologous metastases: demonstration of selective losses of HLA antigens on colorectal, gastric and laryngeal carcinomas

M.A. López-Nevot[1], F. Esteban[2], A. Ferrón[3], J. Gutiérrez[3], M.R. Oliva[1], C. Romero[1], C. Huelin[1], F. Ruiz-Cabello[1] & F. Garrido[1]

[1]*Servicio de Análisis Clínicos,* [2]*Servicio de Otorrinolaringología,* [3]*Servicio de Cirugia General, Hospital Virgen de las Nieves, Granada 18014, Spain.*

**Summary** The expression of HLA class I antigens was studied in 99 primary tumours (colorectal, gastric and laryngeal carcinomas) and 57 autologous metastases using immunohistological techniques and monoclonal antibodies against class I monomorphic determinants, HLA-B isotypic determinants and HLA polymorphic determinants. Fourteen per cent of colorectal, 9.6% of gastric and 20% of laryngeal carcinomas completely lacked class I molecules. Selective losses of HLA-B antigens were also detected in 8.8, 3.4 and 5.8% of these tumours respectively. Taking into account complete and selective loss of HLA-B the average alteration in the class I molecules expression totalled 21%. The comparison of class I expression between primary tumours and autologous metastases showed differences in 24% of the patients. These differences consisted mainly in a decrease of class I expression by metastases. Nevertheless, four types of divergence were detected in laryngeal carcinomas, namely: +/−, +/+, −/+, −/−. In addition, a clear correlation between degree of differentiation and class I expression was observed in laryngeal tumours. Finally, when class I gene RFLPs were compared with DNA from 15 tumours and autologous normal mucosa or peripheral lymphocytes, no differences were detected between these samples.

Major histocompatibility antigens (H-2 in mice and HLA in man) were discovered thanks to their role in alloimmune interactions, generating alloantibodies and alloreactive CTLs when transplants were performed between genetically different individuals (Gorer *et al.*, 1948). However, their physiological function remains obscure. These antigens are controlled by a cluster of genes located in chromosome 17 in mice and chromosome 6 in humans. These genes code for the classical transplantation antigens (class I), the immune response associated antigens (class II) and complement genes (class III) (Dausset, 1981). Class I antigens are cell surface glycoproteins composed of a highly polymorphic heavy chain ($M_r$ 45,000) associated in a non-covalent way to $\beta_2$-microglobulin (Ploegh *et al.*, 1981). Class I molecules are widely distributed and expressed in most, but not all, nucleated cells (Daar *et al.*, 1984). Class II antigens are composed of two glycosylated chains ($M_r$ 32,000 and 29,000 respectively) (Cullen *et al.*, 1974), and are predominantly expressed by cells involved in immunological phenomena (B cells, antigen presenting cells and activated T lymphocytes) (Engleman *et al.*, 1980).

Histocompatibility class I and II antigens are involved in different immunological and probably non-immunological phenomena (Klein, 1986). Cytotoxic T-lymphocytes recognise antigens in association with class I molecules (Zinkernagel & Docherty, 1974), and natural killer cell cytotoxicity has been shown to be inversely correlated with the degree of class I expression (Kärre, 1986). It is therefore becoming evident that the immune response against modified cells, virus infected cells or tumour cells is not only influenced by the nature of the specific antigens but also by the quantity and quality of class I molecules present at the tumour cell surface (Fentestein & Schmidt, 1981, Garrido, 1988).

There is an increasing body of evidence which suggests that MHC class I antigen expression is altered on mouse as well as on human tumours (Fentestein & Garrido, 1986). Two types of alterations have been described: (a) loss of class I antigens (Garrido *et al.*, 1979; Schirrmacher *et al.*, 1981); and (b) expression of aberrant class I-like molecules (Orgad *et al.*, 1985; Garrido *et al.*, 1976*b*; Phillips *et al.*, 1986). Furthermore, some reports indicate that these alterations may play a crucial role in tumour dissemination and metastasis (Eisenbach *et al.*, 1985).

We present data which indicate that:

(*a*) complete loss of HLA expression may occur on colorectal, gastric and laryngeal carcinomas;

(*b*) selective HLA-B losses occurred in three colorectal, two laryngeal and one gastric carcinomas which were previously considered class I positive;

(*c*) differing types of divergence of class I expression were shown by primary tumours and autologous metastases;

(*d*) molecular genetic analysis of class I genes on several tumours indicated no class I gene rearrangement or gross depletion when compared to autologous DNA;

(*e*) finally, a strong correlation between the degree of differentiation and class I expression was found in laryngeal carcinomas.

### Materials and methods

### *Tumour specimens*

Tissue was obtained from patients under the case of the Departments of General Surgery and Otorrhinolaryngology (Virgen de las Nieves Hospital) who had not received radiotherapy and/or chemotherapy before surgery. Histopathological diagnosis was confirmed in paraffin sections. All tissues were snap frozen in liquid nitrogen cooled isopentane after coating with OCT within 1–2 h of removal and stored in liquid nitrogen until sectioning for study. Cryostatic sections measuring 5–7 $\mu$m thick were cut and allowed to dry at room temperature for 14–18 h, after which they were fixed for 10 min in acetone, wrapped in aluminium foil and stored at −40°C until staining. A total of 99 primary tumours (34 colorectal, 31 gastric and 34 laryngeal carcinomas) and 57 autologous metastases were studied.

Correspondence: Federico Garrido, Servicio Análisis Clinicos, Hospital Virgen de las Nieves, Granada 18014, Spain.
Received 28 March 1988, and in revised form, 22 September 1988.

*Monoclonal antibodies (MoAbs)*

The following MoAbs were used: W6/32 against a common class I determinant (A,B,C heavy chains) (Barnstable *et al.*, 1978); JOAN-1 (J. Vives) and α-HLA-B (Burrone *et al.*, 1985) which recognise isotypic determinants in HLA molecules; polymorphic MoAbs obtained from the Xth Histocompatibility Workshop (anti A2, numbers 2020, 2025, 2026 and 2027; anti A11, numbers 2017 and 2045; anti B5, number 2091; anti B14, number 2098; anti B44, number 2097 and anti Bw4, numbers 2102 and 2102); GRH1, against $\beta_2$-microglobulin, and GRT2, a MoAb which reacts against the common leukocytic antigen (CD45), to measure lymphocytic infiltration in tumours (López-Nevot *et al.*, 1986).

*HLA typing*

Peripheral blood lymphocytes were isolated by Ficoll-Hypaque gradient centrifugation (Boyum, 1968) and HLA typed by standard method microcytotoxicity (Terasaki & McClelland, 1964). Tumour HLA typing was performed by immunohistochemical techniques on cryostatic sections (Cordell *et al.*, 1984). The reaction obtained with the infiltrating lymphocytes was used as a positive control in HLA-B negative tumours.

*Alkaline immunophosphatase technique*

Tissue sections were incubated with the first antibody in a moist chamber at room temperature for 45 min. After washing with 0.05 M Tris buffered saline (pH 7.6) (TBS), the sections were incubated for 30 min with rabbit antimouse immunoglobulin at a dilution of 1:20 (DAKO), washed with TBS and then reincubated for 30 min with APAAP complex at a dilution of 1:50 (DAKO). After washing with TBS, fresh chromogen solution was added (0.2 mg $ml^{-1}$ naphthol AS-MX phosphatase, 1 mg $ml^{-1}$ Fast Red salt, 20 μl $ml^{-1}$ dimethylformamide, 0.05 M TRIS buffered saline pH 8.2) (Sigma). A final washing with TBS was followed by counterstaining with Haematoxylin and mounting with Apathy's mounting solution (Cordell *et al.*, 1984).

*DNA isolation and Southern blot analysis*

High molecular weight DNA was isolated from the tumours, normal autologous mucosa and autologous peripheral blood lymphocytes (Blin & Stafford, 1976). Aliquots 10 μg of DNA were digested with 150–300 units of different restriction enzymes (EcoRi, HINDIII, BamHI, PstI, PvuII, MspI or HpaII) for 4 h according to the supplier's specifications (Amersham). DNA fragments were separated by electrophoresis in 0.8% agarose gels for 16 h and transferred to nitrocellulose membranes (Southern, 1975). A 1.4 Kbp cDNA coding for HLA-B7 (pDP-001) was used as a probe (Sood *et al.*, 1981). Membranes were treated for hybridisation with nick-translated P32-labelled pDP-001 in 50% formamide hybridisation solution for 36–40 h at 42°C. Membranes were washed at 65°C for 30 min in two changes of 2 × SSC/0.1% SDS. After washing, the membranes were dried, and autoradiographed at −70°C until the signals were visible.

### Results

*Expression of class I antigens*

*Colorectal carcinomas* We examined the expression of HLA class I antigens on 34 colorectal primary tumours and 13 metastases (12 lymph node and one liver metastases). Twenty-one primary tumours (61.8%) presented a homogeneous pattern of HLA-A, B and C antigen expression. Five tumours also showed a positive but heterogeneous pattern of expression, while five tumours were class I negative (Table I). MoAbs were used against monomorphic determinants of HLA-A, B and C and $\beta_2$-microglobulin. There was no discrepancy among the reaction patterns obtained with these MoAbs. However, differences were observed in three patients (8.8%) when two HLA-B locus specific MoAbs were used. These three patients showed selective losses of HLA-B products.

Samples of normal mucosa from the same patients distal or closer to the tumour were also studied. Benign lesions (adenomas) were also included in the protocol. No alteration in class I expression was observed in these samples. Nor was any correlation found between the partial or complete loss of class I antigen expression and the degree of differentiation of the primary colorectal tumours studied (Table II).

The 13 colorectal metastases studied were class I positive. Nevertheless, eight metastases presented a lower percentage of positive cells than the autologous primary tumours (Table III).

*Gastric carcinomas* Thirty-one primary gastric carcinomas were studied. Class I antigens were completely absent in three patients (9.6%) (Table I). Heterogeneous expression was observed in five patients (16.1%) (between 25 and 75% positive cells). Twenty-two patients (71.1%) showed positive and heterogeneous class I expression. Selective loss of HLA-B locus antigen was found in only one tumour.

HLA class I expression was considerable in 16/19 poorly differentiated gastric carcinomas, whereas only 6/12 well differentiated carcinomas were positive (Table II). Interestingly, normal gastric mucosa showed weak, heterogeneous HLA-A, B and C antigen expression. Thirty lymph node metastases were studied, of which 27 were found to be class I positive and three class I negative (Table III). Hence the vast majority of lymph node metastases derived from gastric tumours were class I positive.

*Laryngeal carcinomas* Thirty-four primary laryngeal carcinomas were studied, seven of which (20.5%) were class I negative. In addition, B-locus losses were observed in two tumours (5.8%) (Table I). The degree of differentiation was

**Table I** HLA class I antigen expression in colorectal, gastric and laryngeal carcinomas

| *Percentage reactivity with MoAbs against* | | *Number of tumours* | | |
|---|---|---|---|---|
| *A,B,C antigens/$\beta_2$-m* | *HLA-B* | *Colorectal* | *Gastric* | *Laryngeal* |
| 100 | 100 | 21 | 22 | 24 |
| 75–50 | 75–50 | 4 | 3 | 0 |
| 50–25 | 50–25 | 1 | 2 | 1 |
| <25 | <25 | 5[b] | 3[b] | 7[b] |
| 100 | <5 | 3[a] | 1[a] | 2[a] |
| Total number of primary tumours | | 34 | 31 | 34 |

[a]Three colorectal (8.8%), one gastric (3.4%) and two laryngeal tumours (5.8%) present selective loss of HLA-B locus products. [b]In 5/34 (14%) of colorectal, 3/29 (9.6%) of stomach and 7/34 (20%) of laryngeal tumours HLA A, B and C antigens were completely absent.

**Table II** Relationship between class I expression on primary tumours and grade of differentiation

| *Class I*[a] | *Colorectal (34)*[b] | | | *Gastric (31)* | | | *Laryngeal (34)* | | |
|---|---|---|---|---|---|---|---|---|---|
| | *I* | *II* | *III* | *I* | *II* | *III* | *I* | *II* | *III* |
| 100 | 8 | 10 | 3 | 1 | 5 | 16 | 8 | 16 | – |
| 75–25 | 1 | 2 | 2 | – | 3 | 2 | – | – | 1 |
| <25 | 1 | 3 | 1 | 1 | 1 | 1 | 1 | – | 6 |
| HLA-B (–) | – | 3 | – | – | 1 | 1 | – | 2 | – |

[a]Percentage of positive cells. [b]Number of cases.
Degree of differentiation: I, well differentiated; II, moderately differentiated; III, poorly differentiated.

**Table III** Expression of class I antigens in lymph node metastases of colorectal, gastric and laryngeal carcinomas

| | *A,B,C antigens/$\beta_2$-m (+/total)* | *HLA-B antigen (+/total)* |
|---|---|---|
| Colorectal | 13/13[a] | 13/13 |
| Gastric | 27/30[b] | 27/30 |
| Laryngeal | 9/14[c] | 9/14 |
| Total number of metastases | 57 | 57 |

[a]All 13 colorectal carcinoma metastases were HLA class I positive. However, in eight cases class I expression was lower than on autologous primary tumours. [b]Three gastric carcinoma metastases were HLA class I negative. [c]Five laryngeal carcinoma metastases were HLA class I negative.

strongly correlated with class I antigen expression. All tumours expressing large amounts of HLA showed a well or moderately differentiated pattern. On the contrary, 6/7 class I negative tumours were poorly differentiated (Table II). It was observed that normal laryngeal mucosa was always class I positive, presenting a homogeneous staining pattern. Fourteen autologous metastases were studied, of which nine were found to be class I positive and five class I negative (Table III).

*Differences in HLA class I expression between primary tumours and autologous metastases*

The class I expression of 57 metastases was compared with that of their autologous primary tumours. In most of the patients there was no detectable difference. In eight patients with colonic carcinoma and two with gastric carcinoma a decrease in the number of HLA class I positive cells was observed on the metastases (Table IV). In six laryngeal carcinomas, four possible combinations of expression between the primary tumour and its autologous metastases (+/+, +/–, –/+, –/–) were found (Table IV).

*Selective loss of HLA-B locus products*

Six of 99 primary tumours (6%) were found to be selective for HLA-B antigen losses (Table I). Considering that the amount of HLA class I losses reached 15% (15/99) we believe that the 6% of B-locus losses should be added to this figure, resulting in a total of 21%.

One case was selected from each tumour group to confirm the HLA-B antigen loss. These three patients were HLA typed using the standard Terasaki microcytotoxicity test on PBLs. Tumour tissue was typed on frozen sections with polymorphic HLA reagents using the immune alkaline phosphatase technique, in conjunction with MoAbs defining polymorphic HLA determinants. These MoAbs were developed at the Xth International HLA workshop and define the following specificities: A2, A11, B5, B14, B21, B44 and Bw4 (Table V).

The loss of B locus products in these tumours was confirmed with polymorphic MoAbs. In all cases positive reaction with the infiltrating lymphocytes was used as the internal control. In one gastric tumour, HLA-A locus antigens were also undetectable despite a positive reaction with W6/32 and $\beta_2$-microglobulin.

**Table IV** Differences in HLA class I expression antigen between primary tumour and autologous lymph node metastases[a]

| | *Primary tumour* | *Autologous metastases* |
|---|---|---|
| LC1 | – | – |
| LC2 | – | +M1[c] |
| | | +M2 |
| LC3 | + | –M1 |
| | | –M2 |
| LC5 | + | + |
| LC7 | + | –M1 |
| | | –M2 |
| GC1[b] | + | – |
| GC2 | + | – |
| CC1[b] | + | + |

(–), Less than 25% of stained cells; (+), more than 25% of stained cells; LC, laryngeal carcinoma; GC, gastric carcinoma; CC, colorectal carcinoma; [a]Fifty-seven metastases were studied. Most of the patients showed no detectable differences when class I antigen expression was compared between primary tumour and autologous metastases. This table illustrates several examples in which differences were found. [b]In eight patients with colorectal and two gastric carcinoma the number of positive cells for class I antigens in the primary tumour was higher than in metastases. [c]In some patients it was possible to compare HLA class I antigen expression among multiple metastases (M1, M2) from the same primary tumour.

**Table V** Selective loss of HLA class I antigen in human tumours

| | *Lymphocytic typing* | *Tumour typing* |
|---|---|---|
| Colon | A2, –/B5, B21 | A2, –/–, – |
| Larynx | A2, –/B5, B44 | A2, –/–, – |
| Stomach | A11, –/B14, B44 | –, –/–, – |

Lymphocytes were tested by standard method microcytotoxicity and tumour typing by immunohistological techniques using polymorphic monoclonal antibodies.

*Comparative study of HLA class I genes from normal tissue and autologous tumours*

Southern blot analysis of class I genes was performed on 15 different tumours (10 colorectal, 2 gastric and 3 laryngeal carcinomas). Normal autologous DNA extracted from normal cells (autologous mucosa or PBL) was used as a control, and pDP-001 was used as a probe. The 15 different tumours selected included seven class I negative, three B-locus negative and five class I positive specimens. No differences were found in RFLPs of these tumours when compared with normal autologous DNA (Figure 2). We also included methylation sensitive enzymes, which failed to detect differences between tumour and normal mucosa DNA.

**Figure 1** Alkaline immunophosphatase stained cryostatic sections of laryngeal carcinoma labelled with W6/32 (**a**) or anti-HLA-B (**b**) monoclonal antibodies. (**a**) W6/32 reacting positively on epithelial and stromal cells in a tumour section. (**b**) Anti-HLA-B MoAb (JOAN-1) reacting negatively in the same tumour. The stromal cells were stained in the same preparation as a control.

**Figure 2** Southern blot analysis of class I genes from colon (**a**), gastric (**b**) and laryngeal (**c**) carcinoma. DNA from the tumour (T) was compared with DNA from autologous normal mucosa (M) or autologous lymphocytes (L). No differences in RFLPs were observed. This probe was a cDNA of HLA-B7 (pDP001).

### Discussion

The phenotypic analysis of HLA class I molecules on colorectal, gastric and laryngeal carcinomas with MoAbs directed against monomorphic determinants reveals three different reaction patterns: positive, heterogeneous and negative.

Heterogeneity of class I expression has previously been detected in breast carcinoma (Natali *et al.*, 1983; Pérez *et al.*, 1986) and in melanoma (Albino *et al.*, 1981). The most widely accepted explanation to date is based on the appearance of clones with differential HLA class I expression in the course of tumour progression (Ruiz-Cabello *et al.*, 1987).

The loss of HLA class I expression is a relatively frequent event in human neoplasias, and has been correlated with the degree of differentiation in embryonic carcinoma (Lampson *et al.*, 1983), histological type in carcinoma of the lung (Doyle *et al.*, 1985), degree of tumour progression in melanoma (Bröcker *et al.*, 1985, López-Nevot *et al.*, 1986) and also with the malignancy of B cell lymphomas (Möller *et al.*, 1987).

Contrary to the findings described by others (Morburg *et al.*, 1986), no correlation between the loss of HLA class I expression and the degree of cell differentiation was found in colorectal carcinoma (Gutiérrez *et al.*, 1987). However, it was noted that in laryngeal carcinomas most of the tumours consisting of less differentiated cells were HLA class I negative (6/7) whereas among moderately or well differentiated carcinomas there was only one HLA class I negative tumour. These data suggest that the loss of HLA class I expression is closely related to the degree of cell differentiation in laryngeal carcinoma. This loss may also represent an escape mechanism from cytotoxic T-cell mediated lysis, as these cells recognise tumour antigens in association with HLA class I molecules (Fentestein, 1987). On the other hand, the loss of HLA class I expression may lead to increased sensitivity of tumour cells to lysis by NK cells (Ljungren *et al.*, 1986). In gastric carcinoma, however, the less differentiated cells are mainly HLA class I positive exhibiting higher levels of expression than those observed in normal gastric mucosa. This increased HLA class I expression may facilitate their metastatic progression by means of diminishing their susceptibility to NK lysis.

When HLA class I expression on primary tumours was compared with autologous metastases in laryngeal carcinoma, some HLA class I positive primary tumours demonstrated HLA class I negative metastases and vice versa. These data suggest that several mechanisms of clonal selection may coexist during the course of tumour progression, given the fact that there does not seem to be a simple pattern of change in HLA class I expression. The differences in HLA class I expression between primary tumours and autologous metastases in colorectal and gastric carcinoma, basically consisting of a decrease in the number of HLA class I positive cells, were observed only in a small number of cases.

Anti-HLA class I MoAb directed against a monomorphic determinant does not effectively detect selective losses of HLA-A, B or C antigen expression. However, when using isotypic MoAbs directed against HLA-B specific determinants it became possible to record losses of HLA-B antigens in all three types of tumour studied. The reactivity of the lymphoid infiltrate with these antibodies served as an internal control. In these three tumours it was also possible to confirm the absence of HLA-B molecules by means of polymorphic MoAbs directed against the serologically detected specificities found in autologous blood lymphocytes by standard cytotoxicity assays. In one case of HLA class I positive gastric carcinoma HLA-B reactivity was not observed and anti-HLA-A MoAb assays were found to be negative. As HLA-C antigens are generally expressed in much lower amounts than HLA-A or B, the antibody W6/32 may, in this case, recognise HLA class I-like molecules which are distinct from HLA-A, B or C.

These data speak in favour of the use of isotypic MoAbs but also polymorphic antibodies in order more accurately to define alterations in HLA class I expression. Even with isotypic MoAbs it would be difficult to detect a partial loss of antigen expression if the defect was located in the polymorphic domains of the molecule. Selective losses of MHC class I antigens have been described in murine tumours (Garrido *et al.*, 1976*b*). These losses of K or D molecules were correlated with an enhanced metastatic ability (Eisenbach *et al.*, 1983). It has recently been reported that the resistance of certain Burkitt lymphomas to lysis by autologous cytotoxic T-lymphocytes may be due to the selective loss of HLA-A11 antigen expression (Masucci *et al.*, 1988). Selective losses of HLA-B locus products have been recently reported in colorectal carcinomas (Garrido, 1988; Smith *et al.*, 1988). The selective loss of HLA-B antigens may give rise to a similar state. It would also be worthwhile to study whether selective losses of HLA class I antigen expression affect certain specificities more frequently than others.

Southern blot analyses were performed to try to ascertain whether the changes in HLA class I expression could be due to a genomic structural alteration (rearrangement or gross deletion) or perhaps to changes in the methylation pattern of the CCGG base sequences (using the isoschizomeric pair of restriction enzymes MspI and HpaII which are differentially sensitive to methylation of the CCGG moiety). Data have been obtained on the murine GR9 tumour which substantiate different class I methylation patterns between class I positive and negative tumour clones (Bonal *et al.*, 1986). The loss of H-2 molecules in SV-40 and radiation leukaemia virus induced tumours is associated with H-2 class I gene rearrangement or CCGG hypermethylation (Rogers *et al.*, 1983, Meruelo *et al.*, 1986). Loss of HLA class I gene restriction fragments has been described in human melanoma (Angelini *et al.*, 1986) and colorectal carcinoma (Bar-Eli *et al.*, 1987). When tumour DNA was compared with that from normal autologous cells it was not possible to detect clear genomic alterations with the enzymes and probe (HLA class I) used. In the present study HLA class I positive and negative tumours as well as tumours with selective losses of HLA-B locus products were investigated. The cause of altered HLA class I expression in our tumours may therefore be mediated by a transcriptional event. Selective losses of HLA molecules without DNA damage suggest that the B-locus can be regulated independently from the A-locus and as similarly described on murine K and D class I molecules (Gmür *et al.*, 1980).

Further studies remain to be carried out in order to analyse the alteration in histocompatibility antigens expression and its role in tumour progression and metastases.

We thank Sir Walter Bodmer, Dr C. Milstein and Dr J. Vives for the provision of monoclonal antibodies, and Dr A. Biro for the class I probes; Miss Karen Shashok for revising the English style; Valeri Verbi and Rosa Fernández for typing the manuscript. These studies were supported by the Fondo Investigaciones Sanitarias Grants 86/1009, 87/1723 and CAICYT grant OA85-0073 (Spain).

### References

ALBINO, A.P., LLOYD, K.O., HOUGHTON, A.N., OETTGEN, H.F. & OLD, L.L.J. (1981). Heterogeneity in surface antigen and glycoprotein expression of cell lines derived from different melanoma metastases of the same patient. *J. Exp. Med.*, **154**, 1764.

ANGELINI, G., FOSSATI, G., RADICE, P. & 5 others (1986). Loss of polymorphic restriction fragments of class I and class II MHC genes in a malignant melanoma. *J. Immunogenol.*, **13**, 241.

BAR-ELI, M., BATTIFORA, H. & CLINE, M.J. (1988). Alterations of class I HLA genes in human colon cancers. *Human Genetics*, **78**, 86.

BARNSTABLE, C.J., BODMER, W.F., BROWN, G. & 4 others (1978). A production of monoclonal antibodies to group A erythrocytes, HLA and other human cell membrane antigens. New tools for genetic analysis. *Cell*, **14**, 9.

BLIN, N. & STAFFORD, D.W. (1976). Isolation of high molecular-weight DNA. *Nucleic Acids Res.*, **3**, 2303.

BONAL, F.J., PAREJA, E., MARTIN, J., ROMERO, C. & GARRIDO, F. (1986). Repression of class I H-2K, H-2D antigens on GR9 methylcholantrene induced tumour cell clones is related to the level of DNA methylation. *J. Immunogen.*, **13**, 179.

BOYUM, A. (1968). Separation of leukocytes from blood and bone marrow. *Scand. J. Clin. Lab. Invest.*, **21**, Suppl. 97, 77.

BRÖCKER, E.B., SUTER, L., BRÜGGEN, J. RUITER, D.J., MACHER, E. & SORG, C. (1985). Phenotypic dynamics of tumour progression in human malignant melanoma. *Int. J. Cancer*, **36**, 29.

BURRONE, O.R., KREFFORD, R.F., GILMORE, D. & MILSTEIN, C. (1985). Stimulation of HLA-A, B, C by IFN. The derivation of Molt 4 variants and the differential expression of HLA, B, C subsets. *EMBO J.*, **4**, 2855.

CORDELL, J.L., FALINI, B., ERBER, W.N. & 6 others (1984). Immunoenzymatic labeling of monoclonal antibodies using immunocomplexes of alkaline phosphatase and monoclonal anti-alkaline phosphatase (APAAP complexes). *J. Histochem. Cytochem.*, **32**, 219.

CULLEN, S.E., DAVID, C.S., SCHREFFLER, D.C. & NATHENSON, S.G. (1974). Membrane molecules determined by the H-2 associated immune response region: isolation and same properties. *Proc. Natl Acad. Sci. USA*, **71**, 648.

DAAR, A.S., FUGGLE, S.V., FABRE, J.V., TING, A. & MORRIS, P.J. (1984). The detailed distribution of HLA ABC antigens in normal human organs. *Transplantation*, **38**, 287.

DAUSSET, J. (1981). The major histocompatibility complex in man. Past, present and future concepts. *Science*, **213**, 1469.

DOYLE, A., MARTIN, V.J., FNA, K. & 8 others (1985). Markedly decreased expression of class I histocompatibility antigen, protein and mRNA in human small-cell lung cancer. *J. Exp. Med.*, **161**, 1135.

EISENBACH, L., FELDMAN, M. & SEGAL, S. (1983). MHC imbalance and metastatic spread in Lewis lung carcinoma clones. *Int. J. Cancer*, **32**, 113.

EISENBACH, L., HOLLANDER, N., SEGAL, S. & FELDMAN, M. (1985). The differential expression of class I major histocompatibility complex antigens controls the metastatic properties of tumour cells. *Transplantation Proc.*, **27**, 729.

ENGLEMAN, E.G., CHARRON, D.J., BENIKE, C.J. & STEWART, G.J. (1980). Ia antigen on peripheral blood mononuclear leukocytes in man. Expression biosynthesis and function of HLA-DR antigen on non-T-cells. *J. Exp. Med.*, **152**, 99s.

FENTESTEIN, H. (1987). The biological consequences of altered MHC expression on tumours. *Br. Med. Bull.*, **43**, 217.

FENTESTEIN, H. & GARRIDO, F. (1986). MHC antigens and malignancy. *Nature*, **332**, 502.

FENTESTEIN, H. & SCHMIDT, V. (1981). Variations in MHC antigen profiles of tumour cells and its biological effects. *Immunol. Rev.*, **60**, 85.

GARRIDO, F. (1988). *H-2 Gene Complex: Genes, Molecules, Functions*. C. David: Bar Harbor, Maine.

GARRIDO, F., FENTESTEIN, H. & SCHIRMACHER, V. (1976*a*). Further evidence for depression of H-2 and Ia-like specificities of foreign haplotypes in mouse tumour cell lines. *Nature*, **261**, 705.

GARRIDO, F., PÉREZ, M. & TORRES, M.D. (1979). Absence of four H-2d antigenic specificities in an H-2d sarcoma. *J. Immunogen.*, **6**, 83.

GARRIDO, F., SCHIRMACHER, V. & FENTESTEIN, H. (1976*b*). H-2-like specificities of foreign haplotypes appearing on a mouse sarcoma after *Vaccinia* virus infection. *Nature*, **259**, 228.

GORER, P.A., LYMAN, S. & SNELL, G.D. (1948). Studies on the genetic basis on tumour transplantation. Linkage between an histocompatibility gene and fused in mice. *Proc. Roy. Soc. B*, **135**, 499.

GMÜR, R., SOLTER, D. & KNOWLES, B.B. (1980). Independent regulation of H-2K and H-2D gene expression in murine teratocarcinoma somatic cell hybrids. *J. Exp. Med.*, **151**, 1349.

GUTIÉRREZ, J., LÓPEZ-NEVOT, M.A., CABRERE, T. & 4 others (1987). Class I and II HLA antigen distribution in normal mucosa, adenoma and colon carcinoma: relation with malignancy and invasiveness. *Exp. Clin. Immunogen.*, **4**, 144.

KÄRRE, K., LJUNGREN, H.G., PIONTEK, G. & KIESSLING, R. (1986). Selective rejection of H-2 deficient lymphoma variants suggests alternative immune defence strategy. *Nature*, **319**, 675.

KLEIN, J. (1986). *Natural History of the Major Histocompatibility Complex*. Wiley: New York.

LAMPSON, L.A., FISHER, C.A. & WHELAN, J.P. (1983). Striking paucity of HLA-A, B, C and $\beta_2$-microglobulin on human neuroblastoma cell lines. *J. Immunol.*, **13**, 2471.

LJUNGREN, H.G. & KÄRRE, K. (1986). Experimental strategies and interpretations in the analysis of changes in MHC gene expression during tumour progression. *J. Immunogen.*, **13**, 141.

LÓPEZ-NEVOT, M.A., GARCÍA, E., PAREJA, E. & 5 others (1986). Differential expression of HLA class I and II antigens in primary and metastatic melanomas. *J. Immunogen.*, **13**, 219.

MASUCCI, M.G., TORSTEINDOTTIR, S., COLOMBANI, J., BROUTBOR, C., KLEIN, E. & KLEIN, G. (1988). Down regulation of class I HLA antigens and of the Epstein-Barr virus (EBV) encoded latent membrane protein (LMP) in Burkitt lymphoma lines. *Proc. Natl Acad. Sci. USA* (in the press).

MERUELO, D., KORNREICH, R., ROSSOMANDO, A. & 9 others (1986). Lack of class I H-2 antigens in cells transformed by radiation leukemia virus is associated with methylation and rearrangement of H-2 DNA. *Proc. Natl Acad. Sci. USA*, **83**, 4504.

MÖLLER, P., HERRMANN, B., MOLDENHAUER, G. & MOMBURG, F. (1987). Defective expression of MHC class I antigens is frequent in B-cell lymphomas of high-grade malignancy. *Int. J. Cancer*, **40**, 32.

MORBURG, F., DEGENER, T., BACCHUS, E., MOLDENHANER, G., HÄMMERLING, G. & MÖLLER, P. (1986). Loss of HLA ABC and de novo expression of HLA DR in colorectal cancer. *Int. J. Cancer*, **37**, 179.

NATALI, P.G., GIACOMINI, A. & BIGOTTI, A. (1983). Heterogeneity in the expression of HLA and tumour associated antigens by surgically removed and cultured breast carcinoma cells. *Cancer Res.*, **43**, 660.

ORGAD, S., YANG, S.Y., GAZIT, E. & 4 others (1985). Expression of extra class I major histocompatibility antigens on T-cell acute lymphoblastic leukemia (ALL) lymphoblasts. *Hum. Immunol.*, **12**, 133.

PÉREZ, M., CABRERA, T., LÓPEZ-NEVOT, M.A., GÓMEZ, M., RUIZ-CABELLO, F. & GARRIDO, F. (1986). Heterogeneity of the expression of class I and II antigens in human breast carcinoma. *J. Immunogen.*, **13**, 247.

PHILLIPS, C., STRAUSS, H.J., WORTZEL, R.D. & SCHREIBER, A. (1986). Novel MHC class I molecule as a tumour-specific antigen: correlation between the antibody defined and the CTL defined target structure. *Immunogenetics*, **13**, 93.

PLOEGH, H.L., ORR, H.T. & STROMINGER, J.L. (1981). Major histocompatibility antigens: the human (HLA A, B, C) and murine (H-2K, H-2D) class I molecules. *Cell*, **24**, 287.

ROGERS, M.J., GODING, L.R., MAGULIES, D.H. & EVANS, G.A. (1983). Analysis of a defect in the H-2 genes of SV40 transformed C3H fibroblast that do not express H-2K. *J. Immunol.*, **130**, 2418.

RUIZ-CABELLO, F., LÓPEZ-NEVOT, M. A. & GARRIDO, F. (1987). MHC class I and II gene expression on human tumours. International Meeting on Cancer Metastases, Bologna.

SCHIRMACHER, V., ROBINSON, P., ALTEVOGT, P. & 5 others (1981). Clonal analysis of H-2 antigen expression by variants of a chemically induced murine tumour. *Transplant. Proc.*, **13**, 1819.

SMITH, M.E.F., BODMER, W.F. & BODMER, J.G. (1988). Selective loss of HLA-A, B, C locus products in colorectal adenocarcinoma. *Lancet*, **i**, 823.

SOOD, A.K., PEREIRA, D. & WEISSMAN, S.M. (1981). Isolation and partial nucleotide sequence of a cDNA clone for human histocompatibility antigen HLA-B by use of an oligonucleotide primer. *Proc. Natl Acad. Sci. USA*, **78**, 616.

SOUTHERN, E.M. (1975). Detection of specific sequences among DNA fragments separated by gel electrophoresis. *J. Mol. Biol.*, **98**, 503.

TERASAKI, P.I. & McCLELLAND, J.D. (1964). A microdroplet assay of human serum cytotoxicity. *Nature*, **204**, 998.

ZINKERNAGEL, R.M. & DOHERTY, P.C. (1974). Restriction of *in vitro* T-cell mediated cytotoxicity in lymphocytic choriomeningitis within a syngeneic semiallogeneic system. *Nature*, **248**, 701.